# Distribution of *Ixodes scapularis* in Northwestern Ontario: Results from Active and Passive Surveillance Activities in the Northwestern Health Unit Catchment Area

**DOI:** 10.3390/ijerph15102225

**Published:** 2018-10-11

**Authors:** Erin Schillberg, Dorian Lunny, L. Robbin Lindsay, Mark P. Nelder, Curtis Russell, Mike Mackie, Dave Coats, Alex Berry, Kit Ngan Young Hoon

**Affiliations:** 1 Northwestern Health Unit, Kenora, ON P9N 2K4, Canada; eschillberg@nwhu.on.ca (E.S.); mmackie@nwhu.on.ca (M.M.); dcoats@nwhu.on.ca (D.C.); aberry@nwhu.on.ca (A.B.); kyounghoon@nwhu.on.ca (K.N.Y.H.); 2 Canadian Public Health Service, Public Health Agency of Canada, Ottawa, ON K1A 0K9, Canada; 3 Zoonotic Diseases and Special Pathogens, National Microbiology Laboratory, Public Health Agency of Canada, Winnipeg, MB R3E 3R2, Canada; robbin.lindsay@canada.ca; 4 Enteric, Zoonotic and Vector-Borne Diseases, Communicable Diseases, Emergency Preparedness and Response, Public Health Ontario, Toronto, ON M5G 1V2, Canada; Mark.Nelder@oahpp.ca (M.P.N.); Curtis.Russell@oahpp.ca (C.R.)

**Keywords:** surveillance, *Ixodes scapularis*, *Borrelia burgdorferi*, Lyme disease, northwestern Ontario

## Abstract

The range of *Ixodes scapularis* is expanding in Ontario, increasing the risk of Lyme disease. As an effective public health response requires accurate information on disease distribution and areas of risk, this study aims to establish the geographic distribution of *I. scapularis* and its associated pathogen, *B. burgdorferi*, in northwestern Ontario. We assessed five years of active and passive tick surveillance data in northwestern Ontario. Between 2013 and 2017, 251 *I. scapularis* were submitted through passive surveillance. The submission rate increased over time, and the proportion infected with *B. burgdorferi* was 13.5%. Active tick surveillance from 2014 to 2016 found few *I. scapularis* specimens. In 2017, 102 *I. scapularis* were found in 10 locations around the city of Kenora; 60% were infected with *B. burgdorferi*, eight tested positive for *A. phagocytophilum*, and one for POWV. *I. scapularis* ticks were found in 14 locations within the Northwestern Health Unit area, with seven locations containing *B. burgdorferi*-positive ticks. We found abundant *I. scapularis* populations in the southern portion of northwestern Ontario and northward expansion is expected. It is recommended that *I. scapularis* populations continue to be monitored and mitigation strategies should be established for rural northern communities.

## 1. Introduction

Lyme disease, caused by the bacteria, *Borrelia burgdorferi* sensu stricto, is the most commonly reported vector-borne disease in North America [1,2]. The bacteria is transmitted in eastern and central North America by the blacklegged tick (sometimes called the deer tick), *Ixodes scapularis* [3]. Lyme disease typically manifests as an influenza-like illness with erythema migrans. However, if left untreated, the bacteria may spread to other tissues and organs, causing severe manifestations involving the skin, nervous system, joints, or heart [4].

The range of *I. scapularis* and consequently the risk of Lyme disease is expanding in Canada [5,6]. The number of documented risk areas has increased from one location in Southern Ontario in the early 1990s [7] to numerous locations across Ontario, Nova Scotia, southeastern Manitoba, and New Brunswick [6]. Ticks are introduced into Canada primarily by migratory birds, which move north from the United States in the spring [8], and are further spread in the surrounding area by mammals, such as white-tailed deer, the preferred host of adult *I. scapularis* [9,10]. In states, like Minnesota, where *I. scapularis* populations have expanded to the international border, white-tailed deer and other large-sized mammals may play a more prominent role in the dispersal of ticks into Canada. After their arrival, climate and landscape/ecological features determine the ability of *I. scapularis* ticks to survive and establish self-sustaining populations. Factors that determine *I. scapularis* survival are the presence of a leaf litter layer to protect from drowning, dehydration, and freezing [5,11], host abundance (i.e., presence of deer and rodents at suitable population densities) [5,12], and climate [5,13]. Increases in annual mean temperature are thought to be the primary factor in the range expansion of *I. scapularis* in southern Canada [14,15]. Climate change is expected to define, in part, the breadth of the tick’s range, as summers become warm enough and long enough to allow life cycle completion and therefore propagation of the species over wider geographic areas of Canada [14,16].

Northwestern Ontario is located directly north of Minnesota, a state where *I. scapularis* have long been established. In addition, northwestern Ontario is along migratory bird routes [17], has an abundance of broad-leaf trees, plentiful white-tailed deer populations, and continuous winter snow cover, which increases soil temperatures despite low air temperatures in the winter [9]. These factors make the region suitable for range expansion of *I. scapularis*. While *I. scapularis* populations have been documented in southern Ontario since the 1970s [7,9], their presence in northwestern Ontario has only been documented since the 1990s [18,19], and an established population was documented in 2006 [20]. Currently, the areas around Kenora and Rainy River are known to have established *I. scapularis* populations [21], and ongoing tick surveillance is in place across the region to monitor the geographic distribution of *I. scapularis* and their associated pathogens, including *B. burgdorferi*.

An effective public health response to Lyme disease requires accurate information on disease distribution and areas of risk to target prevention, education, and resources for effective disease prevention. This is particularly true for emerging diseases, such as Lyme disease, as areas of risk emerge and expand [22]. Therefore, the objective of this study was to establish the geographic distribution of *I. scapularis* and their associated pathogens including *B. burgdorferi*, in northwestern Ontario.

## 2. Materials and Methods

### 2.1. Study Setting

**Figure 1:**
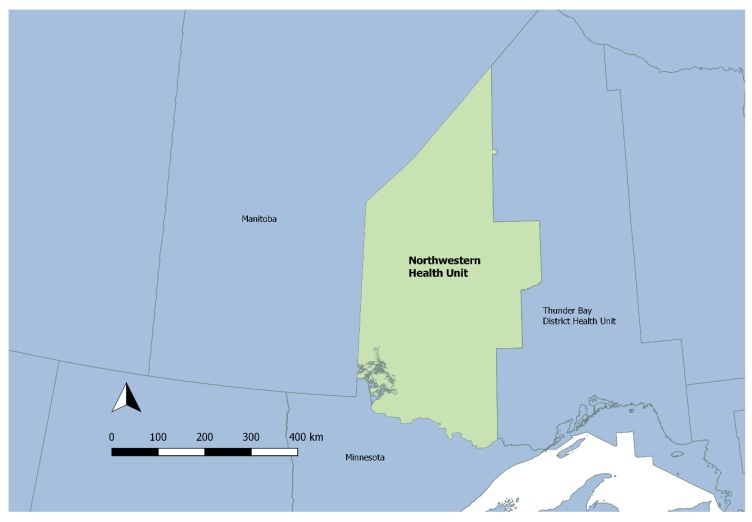
Northwestern Health Unit (NWHU) catchment area map.

The Northwestern Health Unit (NWHU) is the western-most public health unit in the province of Ontario, Canada, bordered by the United States to the South, the province of Manitoba to the West and North, and Thunder Bay District Health Unit to the East (Figure 1). The catchment area covers 173,828 square kilometers, which is about one-fifth of the province of Ontario. The population of 81,886 is dispersed over the region, with a population density of approximately 0.5 people per square kilometer [23]. 

### 2.2. Tick Surveillance

#### 2.2.1. Passive Tick Surveillance

NWHU encourages members of the public within the catchment area to submit tick specimens found on people to any of NWHU’s 12 offices throughout the catchment area. Additionally, physicians encourage their patients who have a blacklegged tick to submit it to NWHU. Ticks from animals are not accepted. Locations of ticks are based on the municipality where the submitter resides, and are not specifically geocoded to the exact location of suspected tick exposure. Any *I. scapularis* that are submitted are sent to the Public Health Ontario Lab (PHOL) for species identification. The PHOL then forwards *I. scapularis* ticks on to the National Microbiology Lab (NML) in Winnipeg for pathogen detection. Tick specimens submitted to the NML were shipped there on freezer packs and held at 4 C prior to DNA extraction and subsequent testing. Test results are shared by NWHU with the client. Details of the passive tick surveillance system in Ontario are described elsewhere [24].

#### 2.2.2. Active Tick Surveillance

Active tick surveillance activities at NWHU are done by public health inspectors (PHIs) who travel throughout the catchment area to conduct tick dragging per Public Health Ontario’s tick dragging standard operating procedure [25]. Active surveillance activities only document *I. scapularis* collected. After dragging the area, any ticks that are captured are sent to the NML for testing and tick specimens were held under the same storage condition as ticks from passive surveillance. Public health inspectors drag for ticks at various locations in the NWHU catchment area in the spring (May and June) and the fall (September and October) each year.

Prior to 2017, dragging locations were selected based on passive surveillance, anecdotal reports, or perceptions of PHIs of areas that might harbor *I. scapularis*. Based on previous years of passive surveillance data, Kenora and the surrounding area was the focus of active surveillance in 2017. Also for this year, the NWHU adopted a more systematic and methodological approach to selecting dragging locations. Using shapefiles obtained from the Ministry of Natural Resources, we mapped out the forest composition of our catchment area using QGIS Version 2.18.13 (QGIS Development Team, Open Source Geospatial Foundation Project) Potential sites to conduct active surveillance in were identified by the presence and quantity of broad-leaf trees (e.g., oak, aspen, poplar), which is a habitat that has been shown to have a positive association with the presence of *I. scapularis* [26]. Forest areas located near accessible roads where the most common type of tree is a broad-leaf were identified as areas to be dragged. 

#### 2.2.3. Pathogen Detection in Blacklegged Ticks

Blacklegged ticks submitted through passive tick surveillance were tested for the presence of *Borrelia burgdorferi*, *Borrelia miyamotoi*, *Anaplasma phagocytophilum*, and *Babesia microti* by real-time PCR as previously described [27]. Ticks submitted in 2017 were also screened for *Borrelia mayonii* [28]. Briefly, QIAGEN DNeasy 96 tissue kits (QIAGEN Inc., Mississauga, ON, Canada) were used for DNA extraction. A duplex screening assay was chosen to screen the samples for *Borrelia* spp. using the 23S rRNA real-time polymerase chain reaction (PCR) assay, and *A. phagocytophilum* using the msp2 real-time PCR assay [29]. Analysis for *B*. *microti* was conducted using the methods described by Nakajima et al. [30] for the detection of the *CCT eta* gene. All *Borrelia* spp.-positive samples were subsequently tested for *B. burgdorferi* using a confirmatory ospA real-time PCR assay, and *B. miyamotoi* using an IGS real-time PCR assay. *B. miyamotoi*-positive samples were further verified using the glpQ real-time PCR assay [27] while *B*. *mayonii* positive samples were confirmed using Light cycler melt curve analysis targeting the *oppA2* gene as described by Pritt et al. [28]. 

To facilitate the detection of RNA viruses in blacklegged ticks, RNA was extracted from individual host-seeking ticks collected by drag sampling in the active tick surveillance program using QIAGEN RNeasy 96 kits (QIAGEN Inc., Mississauga, ON, Canada). Eluate, collected during the RNA extraction of individual ticks, contained both RNA and DNA, and was screened for all the DNA-based pathogens described above. In addition, RNA was also tested for evidence of infection with Powassan virus (POWV) using real-time Reverse Transcriptase (RT)-PCR protocols and primers designed against the *NS5* gene [31]. The lineage of POWV (i.e., lineage 1 or 2) infecting positive ticks was determined using another set of primers against the *NS5* gene as described by Dupuis et al. [31]. To account for possible contamination during extraction and the PCR/RT-PCR procedures, water or blank controls were included in all extractions and PCR/RT-PCR runs, respectively. All of these controls were negative during the course of this study. With the exception of *A. phagocytophilum*, each targeted pathogen was confirmed in ticks using a screening and confirmatory set of primers and probes. 

#### 2.2.4. Data Variables and Sources

Data from *I. scapularis* specimens collected through the passive tick surveillance program between 2013 and 2017 are stored in a secure, internal SharePoint-based database. Variables collected in this database include: the dates of tick collection; date of submission; municipality of collection; tick species submitted; and pathogen infection status.

Data from the active tick surveillance system collected between 2014 and 2017 was collated into one file and used for our analyses. This data file contains the following variables: location of drag sampling; date performed; number; life stage and species of ticks collected; and pathogen infection status.

The relationship between forest composition and *I. scapularis* presence was examined by comparing the average forest composition of areas where *I. scapularis* were found and areas where they were not found. Average forest composition was calculated by determining the average prevalence of each tree species across all sites where *I. scapularis* were found and all sites where they were not found between 2014 and 2017; four forest sections per dragging site were included in these calculations.

### 2.3. Statistical Analysis

Descriptive statistics (numbers, proportions) were used to report results. Linear trends were examined using the Chi-square test for linear trend. The level of significance was set at *p*  ≤  0.05 with 95% confidence intervals. Locations of active tick surveillance dragging sites were geocoded and mapped using QGIS Version 2.18.13. Passive tick surveillance results were geocoded and mapped based on the municipality where the submitter resides. All other analyses were completed using Stata software, version 15 (StataCorp, College Station, TX, USA).

## 3. Results

### 3.1. Passive Tick Surveillance

**Figure 2:**
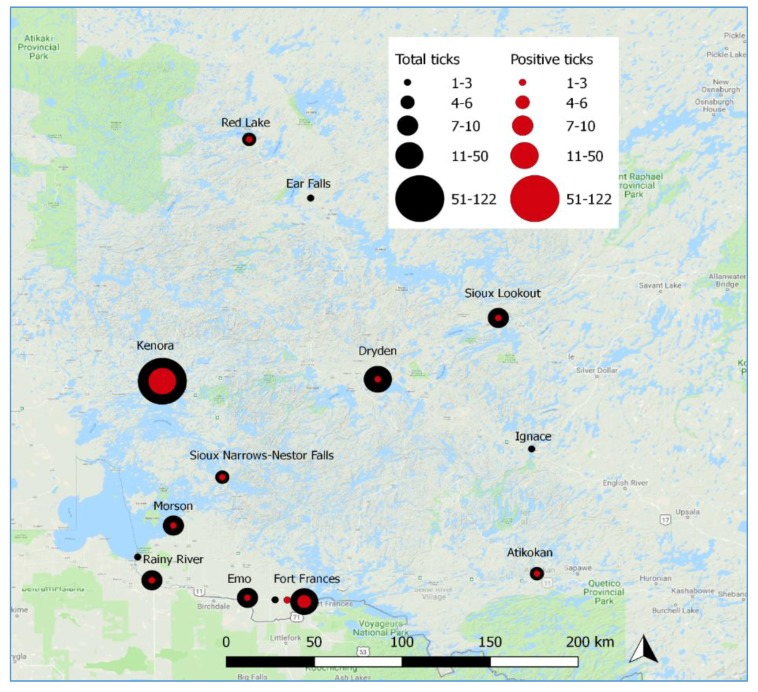
*I. scapularis* submissions through passive tick surveillance by *B. burgdorferi*-positivity in the NWHU catchment area from 2014 to 2017, by location of submission.

From 2013 through 2017, a total of 908 ticks were submitted to NWHU by members of the public. The proportional representation of the different developmental stages was 76.9% female, 15.6% male, and 7.5% nymph. The most commonly-submitted ticks were *Dermacentor variabilis*, followed by *I. scapularis* (Table 1). A total of 251 *I. scapularis* were submitted. There was a general increasing trend in the submission rate of *I. scapularis* submitted each year (chi square for linear trend: 68.417, *p* < 0 .001, Table 2). The proportion of submitted *I. scapularis* infected with *B. burgdorferi* for all five years was 13.5%. No trend over time was observed (Chi square for linear trend: 0.144, *p* = 0.7043, Table 2). The geographic distribution of *I. scapularis* submissions covered a large geographic area, ranging from Red Lake in the northwest to Atikokan in the southeast, and *B. burgdorferi* was detected in blacklegged ticks from almost every site (Figure 2). In the Kenora area in 2017, 9.7% of ticks submitted through passive surveillance were positive for *B. burgdorferi*. Two *I. scapularis* tested positive for *A. phagocytophilum*, both in 2016, submitted by residents of Fort Frances and were negative for *B. burgdorferi.*

### 3.2. Active Tick Surveillance

Active tick surveillance activities from 2014 to 2016 yielded a small number of *I. scapularis* specimens. Four locations were dragged in 2014 yielding a total of four *I. scapularis* that were captured and tested. One tick tested positive for *B. burgdorferi*. Six locations were dragged in 2015 yielding six *I. scapularis* (zero positive). Four locations were dragged in 2016 yielding three *I. scapularis* (zero positive). In 2017, ten locations around the city of Kenora were dragged yielding a total of 102 *I. scapularis*. Sixty-one tested positive, equaling a percent positivity of 59.8% (95% CI: 49.6%, 69.4%; Table 3). In addition, eight *I. scapularis* tested positive for *A. phagocytophilum* and one for POWV (deer tick virus, lineage 2), all in 2017. Six out of the eight ticks that were positive for *A. phagocytophilum* were also positive for *B. burgdorferi* (two females and four males; all found in the Kenora area). The *I. scapularis* that tested positive for POWV was also positive for *B. burgdorferi*.

**Figure 3:**
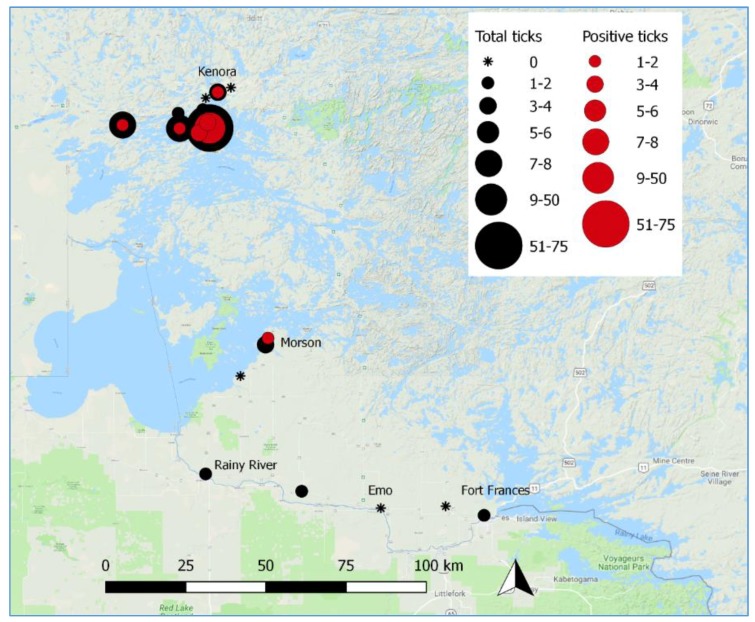
Locations of *I. scapularis* by *B. burgdorferi*-positivity in the NWHU catchment area based on location of active tick surveillance from 2014 to 2017.

All *I. scapularis* captured were adults, with the exception of one nymph that was captured in 2017, which was negative for *B. burgdorferi*. *I. scapularis* were found in 14 unique locations within the NWHU catchment area, with seven of these locations containing *B. burgdorferi*-positive (Figure 3). 

No relationship was found between forest composition and *I. scapularis* presence (Table A1).

## 4. Discussion

Active tick surveillance showed the presence of *I. scapularis* positive for *B. burgdorferi* around Kenora and Morson. As active tick surveillance has been shown to have low sensitivity but good specificity, the capture of an *I. scapularis* by drag sampling indicates an emerging, self-sustaining, reproducing population is present in that location [32]. Adult ticks found by active tick surveillance in these areas had a prevalence of *B. burgdorferi* of about 60%. This is similar to established endemic areas across Michigan (61%) [33]. In addition, an average of two (range: 0–6) human cases of Lyme disease per year between 2011 and 2017 were reported among residents of the NWHU catchment area [34], and previous studies have found an established *I. scapularis* population in the region [20]. This indicates that *I. scapularis* are likely established in the southern portion of northwestern Ontario. On average, over the study period, the percent positivity for passive tick surveillance was 13.5%. Although there was some fluctuation, no significant trend was observed. This was similar to other areas with emerging *I. scapularis* populations, such as Manitoba (9.7%), Quebec (13.0%), and Nova Scotia (15.1%) [35]. However, this is lower than long-established areas, such as Long Point, Ontario (67%) [36]. In Kenora in 2017, 10% of ticks submitted through passive surveillance were positive for *B. burgdorferi*. We speculate that the high percent positivity due to active surveillance in 2017 (60%) could be due to the presence of particular areas within the region that have a large *I. scapularis* population with a high proportion of *B. burgdorferi*. The presence of one of these areas of high endemicity has been previously described in the Kenora area [20].

Failure to detect *I. scapularis* during active tick surveillance along the southern border of the health unit catchment area is likely due to poor selection criteria for sampling sites. The southern border areas are characterized by a higher percentage of agricultural land, making some locations sampled less suitable for ticks. The methodological change in 2017 to sample areas with a high percentage of broad-leaf tree cover (which yielded a much greater prevalence of ticks) was not done along the southern border. Rather, specific sites for active tick surveillance prior to 2017 were chosen based on where members of the public tend to visit (e.g., trails, parks). Furthermore, because of the relatively lower sensitivity of drag sampling, the absence of ticks does not indicate they are not present, but rather they could be present at a density below which they could be detected by active tick surveillance [32]. Passive tick surveillance data also indicate widespread occurrence of *I. scapularis* across NWHU. With an average population density of 0.5 persons/km^2^, it is expected that passive tick surveillance would yield very few submissions [22,30]. However, many ticks were detected across NWHU via passive tick surveillance, leading to the assumption that *I. scapularis* are prominent across the region where the habitat is suitable. 

Passive tick surveillance data indicate that the number of *I. scapularis* in northwestern Ontario is increasing and expanding. This trend has been previously reported in the Rainy River and Kenora areas of northwestern Ontario [21], and in other areas in Canada [6,7,24]. Northward expansion of *I. scapularis* has been observed in Minnesota, with areas of risk reaching the US-Canada border [37]. The increase could be due to the awareness of Lyme disease increasing among Canadians, with government entities producing education campaigns for the public and health care professions [38], leading to increased submission of ticks. Moreover, favorable changes in climate allowing more ticks to survive and establish a population is also a factor in this expansion [6]. By 2080, the average temperatures in northwestern Ontario are expected to increase by 7.0 °C in the northern portion and 6.1 °C in the south [39]. As the climate warms, tick dispersal by birds is expected to occur earlier and cover larger distances [14]. In addition, more adventitious ticks will be able to complete their life cycle and establish self-sustaining populations [14,32]. As *I. scapularis* populations expand across Canada, the proportion carrying *B. burgdorferi* may also increase, raising the risk of Lyme disease among Canadians and pushing this risk further north [24]. Northward expansion to rural and remote communities is a public health concern because, although population densities are low, these areas have limited access to public health and medical services for tick species detection and Lyme disease testing.

We did not find a significant difference in forest composition between areas where *I. scapularis* were found and those were they were not. This is likely explained by the fact that the selection of dragging locations was not originally designed for research purposes. As such, a control group of locations where we would expect to find no *I. scapularis* was not included and as a result most of our dragging locations had relatively similar forest compositions.

Lyme disease and the prevalence and patterns of *I. scapularis* in northwestern Ontario remain issues of public health importance. This indicates the ongoing need for disease and vector surveillance, public education on the disease and prevention measures, and support for health care providers as it relates to diagnosis and treatment. Passive surveillance will continue to be important to direct the health unit towards possible newly emerging *I. scapularis* populations as our climate continues to warm, while active surveillance will be important in the determination of specific risk areas for Lyme disease. Climate change adaptation and vulnerability assessments will be an important tool for health units to assess risks, such as Lyme disease, and establish targeted adaptation strategies. This includes establishing or adapting existing mitigation strategies for rural northern communities. For example, NWHU recently launched a mobile application, which allows individuals to submit photos of ticks to the health unit for identification, broadening our services to areas without public health offices. 

There are some limitations associated with this study. Passive tick surveillance locations are logged based on where the submitter resides, and, therefore, may not capture the exact location of exposure. Active tick surveillance methodology has changed over time, limiting any trend information over time. In addition, the vast geographic area of the health unit could not be systematically covered by active surveillance due to time and resources available. Therefore, only a limited number of sites were surveyed across the region. While we were unable to detect a correlation between forest composition and the presence and abundance of *I. scapularis* ticks, a similar analysis should be completed comparing *I. scapularis* populations across areas of differing forest consumption.

## 5. Conclusions

Our study indicates that populations of *I. scapularis* are well entrenched in the populated regions of NWHU and will likely continue to expand north as the climate continues to change. Vigilance by the public and health care professionals is required to minimize the impact of tick-borne diseases in this region of Ontario.

## Figures and Tables

**Table 1:** Total tick submissions by species submitted through passive tick surveillance in the NWHU catchment area from 2013 to 2017.

Species	2013	2014	2015	2016	2017	Total
*Dermacentor*						
*D*. *variabilis*	35	39	72	201	265	612
*D*. *albipictus*	0	0	0	0	1	1
*Ixodes*						
*I. scapularis*	14	39	17	109	72	251
*I*. *cookei*	2	0	1	12	2	17
*I*. *muris*	0	1	0	0	1	2
Unknown *	2	3	0	0	0	5
*Amblyomma*						
*A*. *cajennense*	1	0	0	0	0	1
Unknown *	0	0	4	9	6	19

* Species not specified.

**Table 2:** *I. scapularis* submission rates per 100,000 population and *B. burgdorferi* status submitted through passive tick surveillance in the NWHU catchment area from 2013 to 2017.

Year	Total *I. scapularis* Submissions *n*	Submission Rate Per 100,000 Population % (95% CI)	*I. scapularis* Positive for *B. burgdorferi* % (95% CI)
2013	14	17.2 (9.4–28.9)	21.4 (4.7–50.8)
2014	39	47.9 (34.1–65.5)	7.7 (1.6–20.9)
2015	17	20.9 (12.2–33.5)	5.9 (0.1–28.7)
2016	109	133.7 (109.8–161.2)	15.6 (9.4–23.8)
2017	72	88.1 (68.9–11.1)	13.9 (6.9–24.1)
Total	251	61.6 (54.2–69.7)	13.5 (9.6–18.4)

**Table 3:** *I. scapularis* submissions by sex submitted through active tick surveillance in the NWHU catchment area from 2017 *.

Pathogen	Male *n* = 54	Female *n* = 47
*B. burgdorferi*	37 (68.5%)	24 (51.1%)
*B. miyamotoi*	0 (0.0%)	0 (0.0%)
*B*. *microti*	0 (0.0%)	0 (0.0%)
*A. phagocytophilum*	5 (9.3%)	3 (6.4%)
*Powassan virus*	0 (0.0%)	1 (2.1%)

* No *I. scapularis* tested positive before 2017, with the exception of one additional *I. scapularis* of unknown sex tested positive for *B. burgdorferi* in 2014.

## Appendix A

**Table A1:** Average forest composition of active surveillance sites in the NWHU catchment area, 2014–2017.

	Average % of Forest Composition	
Tree Species	Sites Where *I. scapularis* Were Detected	Sites Where No *I. scapularis* Were Detected
Trembling Aspen	37.3%	43.0%
White Birch	8.8%	8.0%
Bur Oak	3.3%	0.0%
Black Ash	11.9%	10.0%
Jack Pine	2.3%	4.5%
Black Spruce	5.0%	15.0%
White Pine	4.6%	0.0%
Balsam Fir	8.8%	5.5%
Balsam Poplar	6.3%	8.0%
